# Mouse brain expression patterns of *Spg7*, *Afg3l1*, and *Afg3l2 *transcripts, encoding for the mitochondrial *m*-AAA protease

**DOI:** 10.1186/1471-2202-11-55

**Published:** 2010-04-28

**Authors:** Tiziana Sacco, Enrica Boda, Eriola Hoxha, Riccardo Pizzo, Claudia Cagnoli, Alfredo Brusco, Filippo Tempia

**Affiliations:** 1 Section of Physiology of the Department of Neuroscience, University of Torino and National Institute of Neuroscience-Italy, Torino, Italy; 2 Department of Genetics, Biology and Biochemistry, University of Torino, and S.C.D.U. Medical Genetics, Az. Osp. Univ. San Giovanni Battista, Torino, Italy

## Abstract

**Background:**

The *m*-AAA (**A**TPases **A**ssociated with a variety of cellular **A**ctivities) is an evolutionary conserved metalloprotease complex located in the internal mitochondrial membrane. In the mouse, it is a hetero-oligomer variably formed by the *Spg7*, *Afg3l1*, and *Afg3l2 *encoded proteins, or a homo-oligomer formed by either Afg3l1 or Afg3l2. In humans, *AFG3L2 *and *SPG7 *genes are conserved, whereas *AFG3L1 *became a pseudogene. Both *AFG3L2 *and *SPG7 *are involved in a neurodegenerative disease, namely the autosomal dominant spinocerebellar ataxia SCA28 and a recessive form of spastic paraplegia, respectively.

**Results:**

Using quantitative RT-PCR, we measured the expression levels of *Spg7*, *Afg3l1*, and *Afg3l2 *in the mouse brain. In all regions *Afg3l2 *is the most abundant transcript, followed by *Spg7*, and *Afg3l1*, with a ratio of approximately 5:3:1 in whole-brain mRNA. Using *in-situ *hybridization, we showed that *Spg7*, *Afg3l1 *and *Afg3l2 *have a similar cellular pattern of expression, with high levels in mitral cells, Purkinje cells, deep cerebellar nuclei cells, neocortical and hippocampal pyramidal neurons, and brainstem motor neurons. However, in some neuronal types, differences in the level of expression of these genes were present, suggesting distinct degrees of contribution of their proteins.

**Conclusions:**

Neurons involved in SCA28 and hereditary spastic paraplegia display high levels of expression, but similar or even higher expression is also present in other types of neurons, not involved in these diseases, suggesting that the selective cell sensitivity should be attributed to other, still unknown, mechanisms.

## Background

Mitochondrial AAA-proteases (**A**TPases **A**ssociated with a variety of cellular **A**ctivities) are evolutionary conserved ATP-dependent metalloproteases that participate in the assembly of the respiratory chain complexes and ribosomes, and in mitochondrial protein quality control [1-3].

In mice, two oligomeric AAA-protease complexes are present in the mitochondria inner membrane: the *i*-AAA protease, formed by the YME1L1 protein, oriented towards the intermembrane space, and the *m*-AAA protease, composed by paraplegin (coded by *Spg7*), Afg3l1 and Afg3l2, which exposes its catalytic site to the matrix. It has been recently shown [4] that the *m*-AAA-protease formed by paraplegin has a hexameric structure like FtsH, a better studied bacterial homologue of eukaryotic *m*-AAA members [for review see [5]]. In humans, *SPG7 *and *AFG3L2 *only are active genes, whereas *AFG3L1 *has become a pseudogene. Both *SPG7 *and *AFG3L2 *have been associated with a human neurodegenerative disease: *SPG7 *loss-of-function mutations cause an autosomal recessive form of hereditary spastic paraplegia (HSP) [6], in which axons of corticospinal neurons and of somatosensory neurons degenerate [7]; *AFG3L2 *missense mutations have been implied in the autosomal dominant spinocerebellar ataxia SCA28 [8]. A mouse model for paraplegin deficiency has a motor phenotype that appears at 4 months, and later shows clear features of axonal swelling and degeneration of motor descending and sensory axons [9]. Recently two murine models for *Afg3l2 *have been described, one carrying a null mutation, and a second with a missense mutation in the protease domain. Afg3l2 defective mice display a marked impairment of axonal development leading to lethality at P16 [10]. Mice homozygous for *Spg7 *deletion, which in addition also bear the loss of function *Afg3l2*^Emv66 ^mutation in one allele, display a more severe phenotype with an earlier onset of symptoms when compared with *Spg7 *null mice, and they also develop a marked cerebellar ataxia [11,12].

In yeast, Yta12 and Yta10 are the functional orthologues of paraplegin and AFG3L2, since respiratory competence defects induced by deficiency of both Yta12 and Yta10 are compensated by coexpression of paraplegin and AFG3L2 [13]. Interestingly, the expression of Afg3l2 is sufficient to rescue respiratory competence in this model while paraplegin alone fails to restore *m*-AAA function, in line with the finding that Afg3l2 can form homo-oligomers while paraplegin does not [14].

Recently the assembly of paraplegin in *m*-AAA complexes has been studied in paraplegin deficient HSP fibroblasts and in fibroblast from *Spg7 *-/- mouse model. Different amounts of paraplegin, Afg3l1, and Afg3l2 proteins were seen in liver and brain, with Afg3l1 being the less abundant in the latter. The subunit composition of the proteolytic complex seems therefore to vary in different tissues, and possibly within different subregions [14].

It is therefore interesting to evaluate the relative expression levels of the three murine *m*-AAA components in the brain to further correlate their abundance with the neurodegenerative pathologies associated with *SPG7 *and *AFG3L2 *genes in humans. To this aim, we measured the relative expression of *m*-AAA genes by quantitative reverse-transcriptase polymerase chain reaction (RT-PCR) in the whole brain and in specific regions (cerebellum, hippocampus, neocortex, olfactory bulb); furthermore, a detailed pattern of expression was studied by *in-situ *hybridization under conditions which allowed each probe a sufficient resolution between neurons with different degrees of expression. The combination of RT-PCR quantification with *in-situ *hybridization data allows us to make a reliable comparison of the expression levels of the *m*-AAA transcripts for the first time.

## Results

### In-situ hybridization analysis of m-AAA protease transcripts in the mouse brain

To study the expression of *m*-AAA genes at a single cell resolution, we used non-radioactive *in-situ *hybridization. The results of this analysis for the most relevant brain regions are reported in Table 1. It should be noted that the scoring of expression levels with *in-situ *hybridization was relative to the highest intensity of labeling obtained by each probe, so that the levels can be compared only within each gene but not between genes. The ratio of expression levels across the three genes can be derived taking into account at the same time *in-situ *hybridization data together with quantitative RT-PCR results (see the next section of results).

**Table 1:** Expression patterns of *m*-AAA genes in mouse brain.

	*Spg7*	*Afg3l1*	*Afg3l2*
**Olfactory bulb**			
Glomerular layer	+	++	+
Mitral cell layer	++	+++	+++
Granular cell layer	+/-	+/-	+/-
			
**Hippocampus**			
Dentate gyrus	++	++	+++
Hilus interneurons	+	+	+++
Pyramidal layer	++	++	+++
Subiculum	++	++	++
Stratum radiatum interneurons	+	+/-	+++
Stratum oriens interneurons	+	+/-	+++
Stratum Lac-Mol interneurons	+	+/-	++
			
**Neocortex**			
L 1	+/-	+/-	++
L 2	++	++	++
L 3	++	++	++
L 4	++	++	++
L 5	++	++/+++	+++
L 6	++	++	++
			
**Striatum**	+	+	+
			
**Thalamus**	++	++	++
			
**Cerebellum**			
Purkinje cells	+++	+++	+++
Molecular layer	-	-	-
Granules	+	++	+
Golgi cells	+++	+++	+++
DCN	+++	++	+++
			
**Brainstem**			
Vestibular n.	++	++	++
Pontine n.	-	++	++/+++
Motoneurons of III, V and VII n.	+++	+++	+++
Cuneate n./Gracile n.	++	++	+

Intensity of labeling of *in-situ *hybridization: - indicates absence of labeled structures; + mild labeling; ++ moderate labeling; +++ intense labeling. The scoring was relative to the highest intensity of each probe, so that the levels can be compared only within each gene but not between the three genes analyzed.

**Figure 1:**
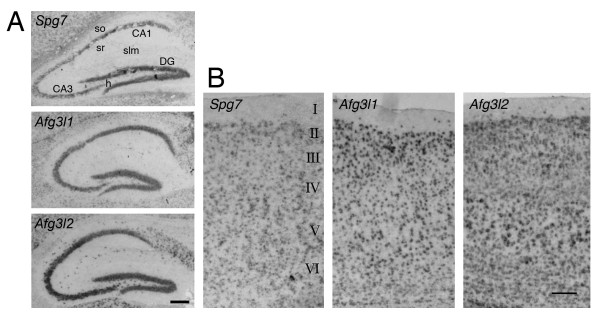
***In-situ* hybridization  staining for *Spg7*, *Afg3l1* and *Afg3l2* in hippocampus and neocortex.** A. Hippocampus. Pyramidal neurons and dentate gyrus are labelled with all three antisense probes. Note that interneurons selectively express *Afg3l2*. Abbreviations: so, stratum oriens; sr, stratum radiatum; slm, stratum lacunosum-moleculare; h, hilus; DG, dentate gyrus; CA1, CA3, hippocampal regions cornu ammonis 1 and 3. B. *In-situ *hybridization staining for *Spg7, Afg3l1 *and *Afg3l2 *in neocortex. Sections of neocortex are shown from the pial surface (top) to the border between layer VI and white matter (bottom). With the exception of layer I, which contains only few cells, all layers display a homogeneous distribution of labelled cells, with a higher staining of layer V. In the left panel, cortical layers are labelled from I to VI. Calibration bars are 125 μm.

We noted a wide variability of expression. A virtual absence of labeling was found in the neurons of the cerebellar cortex molecular layer, whereas a very intense labeling was present in the cerebellar Purkinje cells and in brainstem motor neurons. In the olfactory bulb, the expression was intense in the principal cells of the mitral cell layer, while the glomerular layer showed a moderate expression and the granule cell layer a barely detectable signal. Granule neurons of the dentate gyrus and pyramidal neurons of all hippocampal regions showed a moderate to high expression of all transcripts (Fig. 1A). In contrast to the other two genes, *Afg3l2 *was also clearly expressed by hippocampal interneurons, including those located in the hilus, in stratum oriens, in stratum radiatum and in stratum lacunosum-moleculare (Fig. 1A, bottom panel).

All layers displayed a moderate to intense labeling in neocortex (Fig. 1B), including sparse cells present in the most superficial layer, more abundant with *Afg3l2*. The most salient difference of expression was a more pronounced labeling of layer 5, especially for *Afg3l2*. The level of expression was low in the striatum while in the thalamic nuclei it was moderate.

**Figure 2:**
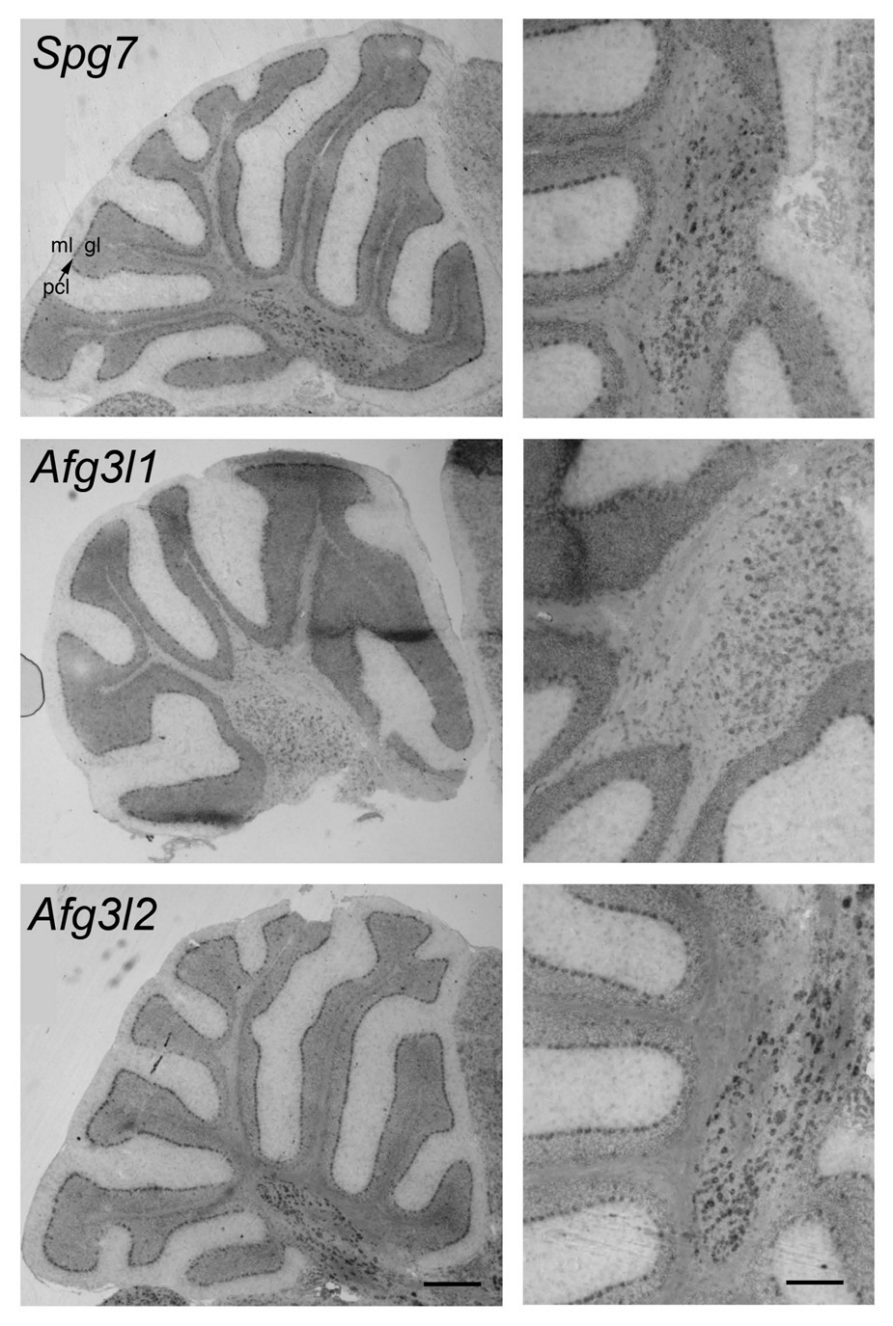
***In-situ *hybridization staining for *Spg7, Afg3l1 *and *Afg3l2 *in cerebellum**. Panels on the left show overviews of cerebella cut along sagittal planes. Panels on the right show the central portion of cerebella at a higher magnification. Note the intense staining of Purkinje cells and of neurons of deep cerebellar nuclei. The molecular layer is devoid of labeling. Abbreviations: ml, molecular layer; gl, granular layer; pcl and arrow, Purkinje cell layer. Calibration bars are 250 μm for left column and 125 μm for right column panels.

In the cerebellum (Fig. 2; see also Additional file 1, panel B, left micrographs), the molecular layer was devoid of labeling, Purkinje cells were very intensely stained and in the granule cell layer middle-sized neurons, presumable Golgi cells, displayed a very high expression, which contrasts with the low labeling of the small sized granule cells. It is noteworthy that also neurons in the deep cerebellar nuclei were densely labeled, especially for *Spg7 *and *Afg3l2*.

In the brainstem, vestibular nuclei expressed all three genes at a moderate level. Pontine nuclei, one of the main sources of mossy fiber afferents to the cerebellum, were not stained for *Spg7*, but had a moderate to high expression of *Afg3l1 *and *Afg3l2*. The neuronal type, which displayed the highest levels of expression of all three transcripts in the brainstem, were motor neurons of cranial nerves, including those located in the nuclei innervating oculomotor muscles (III, IV and VI), trigeminal motor nuclei (V) and facial motor nuclei (VII). Somatosensory cuneate and gracile nuclei also expressed the three transcripts, at a slightly lower level than brainstem motor neurons.

### Quantitative expression of m-AAA protease transcripts in mouse brain regions and in Purkinje cells

**Figure 3:**
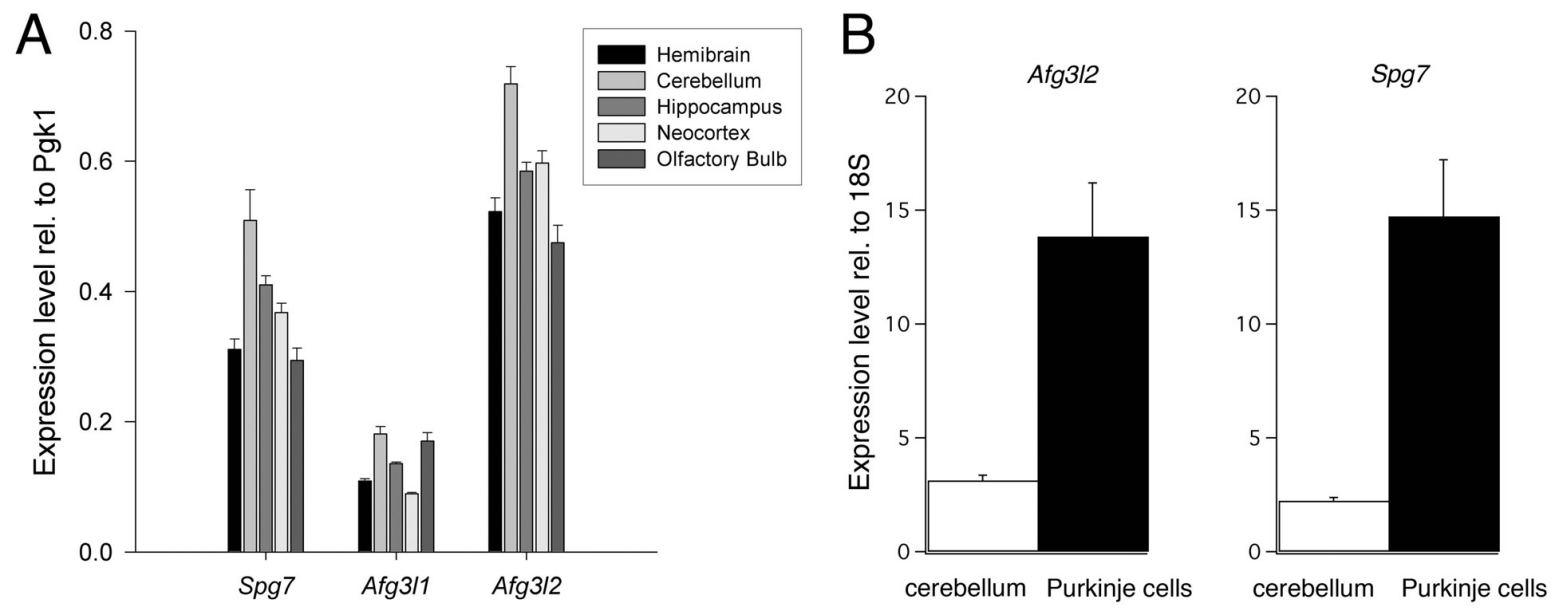
**Levels of expression of *Spg7, Afg3l1 *and *Afg3l2***. A. Real time RT-PCR measures, calibrated relative to the housekeeping gene *Pgk1*. Bars represent standard errors. B. Real-time RT-PCR analysis of *Afg3l2 *and *Spg7 *transcripts in the whole cerebellum and in pools of Purkinje cells. The expression levels are relative to the *18S *rRNA.

The relative expression levels of *Spg7*, *Afg3l1 *and *Afg3l2 *were measured by real-time RT-PCR in the mouse whole brain and in specific regions manually dissected (see methods for details). Data were calibrated using the housekeeping gene *Pgk1 *[15]. In the whole brain, *Afg3l2 *had an expression level, relative to *Pgk1*, of 0.523 ± 0.021, *Spg7 *of 0.311 ± 0.016 and *Afg3l1 *of 0.109 ± 0.003. In all analyzed regions, *Afg3l2 *was always found to be the most abundant transcript, *Afg3l1 *the least expressed, whereas *Spg7 *displayed an intermediate level of expression (Fig. 3A).

For all transcripts we found the highest levels in the cerebellum (Fig. 3A). Besides this region, the neocortex had an expression of *Spg7 *and *Afg3l2 *higher that the average of the whole brain, but showed the smallest amount of *Afg3l1 *transcript (Fig. 3A). These results indicate the presence of some regional differences in the expression of the *m*-AAA genes. The quantification was very reproducible across individuals, indicating a stable area-specific expression of *m-*AAA protease transcripts.

In the cerebellum the prevalent population of neurons are granule cells, which express low levels of *Afg3l2 *and *Spg7*. Since it has been recently reported that *AFG3L2 *is involved in the SCA28 spinocerebellar ataxia [8], in which it is likely that Purkinje cells are affected, it is interesting to quantify the relative level of expression of these cells relative to the average cerebellar expression. In the whole cerebellum (Fig. 3B), the expression level, relative to a housekeeping gene (*18S *rRNA), was 3.13 ± 0.23 for *Afg3l2 *and 2.23 ± 0.15 for *Spg7*. In order to analyze the level of expression in Purkinje cells extracted from slices of cerebellar tissue, the cDNA contents of 10 Purkinje cells were pooled together in order to obtain a constant and reliable amplification. In Purkinje cells (Fig. 3B) the expression of both genes was more than fourfold relative to the cerebellar average, yielding 13.84 ± 2.35 for *Afg3l2 *and 14.73 ± 2.49 for *Spg7*, which are significantly higher than for the whole cerebellum (Student's t-test, *P *= 0.002 for both). This analysis was not extended to *Afg3l1 *because the lower levels of expression of this gene precluded a reliable quantification in Purkinje cells.

## Discussion

Mutations in the mitochondrial *m*-AAA protease genes cause two different neurodegenerative diseases in humans: loss of function of *SPG7 *is associated with an autosomal recessive form of spastic paraplegia [6], whereas missense mutations in *AFG3L2 *have recently been associated with SCA28 [8].

The expression of the three *m*-AAA genes (*Spg7, Afg3l1*, and *Afg3l2*), evaluated by *in-situ *hybridization in the mouse brain, was nearly ubiquitous; only a few structures displayed an almost complete lack of expression, like the molecular layer of the cerebellar cortex. Such a diffuse presence in neurons is in agreement with an essential role for the *m*-AAA complex for neuronal function. An enriched expression of *m*-AAA genes was noted for neurons with large cell bodies, like mitral cells of the olfactory bulb, hippocampal and neocortical pyramidal cells, Purkinje and deep nuclei cells in the cerebellum, and brainstem motor neurons. A similar pattern has been reported for other mitochondrial proteins, and it might be related to a higher requirement for mitochondrial function in large sized neurons [16].

A relative quantification of gene expression between the three *m*-AAA genes was performed by real-time RT-PCR, given that the *in-situ *hybridization technique only allows us to compare the expression of each gene in different cell types. In the whole brain, the proportion of *Afg3l1: Spg7: Afg3l2 *mRNA was about 1:3:5, as suggested by previously reported Western blot experiments [14]. The Allen Brain Atlas http://mouse.brain-map.org/ contains information consistent with our results, but with a faint labeling for *Spg7 *and without the possibility to directly compare the levels of expression of the three *m*-AAA genes. Our real-time RT-PCR data are in agreement with a lower expression of *Spg7 *relative to *Afg3l2*, but with a moderate ratio of 3:5, in accordance with our *in-situ *hybridization data. Furthermore, the moderate to high expression of *Spg7 *in cortical neurons and in motorneurons is more in line with the neuronal damage observed in *Spg7 *null mice [9] and in paraplegin deficient patients [7]. Our data, combining RT-PCR quantification with *in-situ *hybridization, provide the first reliable comparison of the expression levels of the *m*-AAA transcripts, which is not directly possible with either technique alone.

In our real-time RT-PCR study, *Afg3l2 *was the most expressed *m*-AAA gene in all analyzed brain regions, in agreement with the result that Afg3l2 can form both hetero-complexes with paraplegin or Afg3l1, and homo-oligomers [14]. Overall, the higher abundance of Afg3l2 suggests a more important role for this protein than for Spg7 and Afg3l1. Indeed, mice lacking functional Afg3l2 display severe motor deficits, leading to paraparesis and tetraparesis at P12-14, followed by death within P16 [10]. A marked reduction of axonal diameter of cortico-spinal and peripheral nerve fibers is the most striking alteration accompanied by signs of axonal damage and demyelination. The activity of mitochondrial respiratory complex I and III is reduced, and the cytoplasm of motorneurons, dorsal root ganglia and cerebellar Purkinje cells contains clusters of clear vacuoles of possible mitochondrial origin.

*Afg3l1*, a pseudogene in humans, is expressed at low levels in all brain regions analyzed by quantitative RT-PCR. This low level of expression and the non complete overlap of expression pattern with the other *m*-AAA genes, may explain why in both *Spg7 *and *Afg3l2 *knock-out mouse models Afg3l1 is not able to complement the lack of any of the two paralogues [9,10]. We also suggest that the presence of *Afg3l1 *should not be relevant in mouse models for SCA28, where a knock-in *Afg3l2 *is necessary, due to the fact that all patients reported carry a missense mutation [[8]; Cagnoli *et al*., in preparation].

*Spg7 *and *Afg3l2 *are expressed in the cell types related to HSP and SCA28, respectively: *Spg7 *is highly expressed in neocortical pyramidal cells and in motor neurons, and *Afg3l2 *is highly expressed in cerebellar Purkinje cells. Despite the fact that, in Purkinje cells, *Spg7 *is expressed at similar levels as *Afg3l2*, in SCA28 the function of mutated AFG3L2 cannot be substituted by paraplegin, in line with the inability of paraplegin to form homo-oligomers [14]. The high expression of *Afg3l2 *in brainstem motor neurons may also relate to the ophthalmoparesis, which is often present in patients with SCA28 [17]. However, we found that *m*-AAA genes are also expressed at high levels in cell types not directly involved in the corresponding human pathology, suggesting pathogenetic mechanisms not simply based on reduced function but on more subtle alterations.

## Conclusions

In the mouse brain, *Afg3l1: Spg7: Afg3l2 *are expressed at relative levels of 1:3:5. The expression is high in neurons, which are affected in neurodegenerative pathologies associated with *SPG7 *and *AFG3L2 *genes in humans. However, taken together, our results indicate that the neurons affected in HSP and SCA28 do not express selectively *Spg7 *or *Afg3l2 *respectively, nor show the highest brain levels of their relevant transcripts. This suggests that the reason of the cell-specific effect of *Spg7 *or *Afg3l2 *mutations cannot be attributed to selective or particularly high expression levels in a given cell type, but to other still unknown mechanisms.

## Methods

### In-situ hybridization of mouse brain

Non radioactive *in-situ *hybridization was performed on brains collected from adult mice of the the outbred CD-1 line, of 6 months of age (Harlan, Corezzana, Italy). The mice were anaesthetized with isoflurane (Isoflurane-vet, Merial, Milan, Italy) and decapitated. Animal procedures were approved by the Animal Care and Use Committee of the University of Turin. Upon sacrifice, their brains were embedded in OCT (VWR International LTD, Poole, England) and immediately frozen in -80°C 2-methylbutane. Sections (15 μm) were cut by a cryostat (Leica Microsystems GmbH, Wetzlar, Germany) and mounted on SuperFrost Plus glass slides (Menzel GmbH&Co KG, Braunschweig, Germany). All steps were performed at room temperature, unless otherwise specified. Sections were fixed in 4% paraformaldehyde (Merck KGaA; Darmstadt, Germany) in PBS for 10 minutes, and then permeabilized with 0.5% Triton X-100 (Merck KGaA; Darmstadt, Germany) in PBS. An acetylation step was performed using a solution containing triethanolamine and acetic anhydride (both from Merck KGaA; Darmstadt, Germany). After washing with PBS, a pre-hybridization reaction was performed in a solution containing 50% formamide (Euroclone Ltd., Pero, Italy), 2% Blocking Reagent (Roche Diagnostic Corporation, Indianapolis, IN, USA) and 5× SSC. Before the hybridization step, probes were incubated at 85°C for 5 minutes and then on ice, in order to be sure that they were unfolded. We used a set of digoxigenin-labeled riboprobes that span the transcripts of *Spg7, Afg3l1 *and *Afg3l2 *genes; each riboprobe was transcribed in the presence of T3 or T7 RNA polymerase from the appropriately restricted Bluescript plasmid containing the gene fragment of interest (see Additional File 2 probe sequence).

To verify that the probes used in our *in-situ *experiments were specific, we performed a slot blot assay. We prepared three filters each containing three plasmid clones with the full length cDNA of *Spg7*, *Afg3l1 *and *Afg3l2 *murine genes (IRCKp5014I0315Q, IRAKp961C11116Q, and IRAVp968B08129D6, Imagenes). After denaturation with NaOH 0.5 M, EDTA 10 mM, we spotted one-hundred nanograms of each clone onto a N+ nylon filter (Amersham-Pharmacia) using a slot blot apparatus (Biorad). The DNA was covalently bound to the membrane using a UV stratalinker (Stratagene), and hybridised using the Quick-Hyb hybridisation solution (Roche Diagnostics) in the condition specified. Probes were obtained from PCR amplification and gel-purification using QIAquick gel extraction kit (Qiagen), and corresponded in sequence to the probes used for *in-situ *experiments. The probes were radiolabelled with 32-P-dCTP using High Prime labelling kit (Roche Diagnostics). The filters were exposed for 15 min at room temperature. The results [Additional file 1, panel A] show that all probes are specific and only recognize the full length cDNA of the corresponding gene. A very faint cross-hybridisation with *Spg7 *is present for the *Afg3l1 *probe.

Each riboprobe was transcribed as antisense, which specifically labeled the target mRNA as expected, and as sense, to control for unspecific binding or for background staining due to the endogenous alkaline phosphatase activity of the tissue. Examples of sense and antisense labeling are illustrated in panel B of the supplementary fig. [Additional file 1, panel B].

Twenty nanograms of each probe were added to the slices and the hybridization was carried out at 65°C overnight. Washing steps included incubations in 5× SSC and in 0.2× SSC at 65°C for one hour, followed by 5 minutes in 0.2× SSC and 5 minutes in MABS at room temperature. Sections were incubated in 1% Blocking Reagent (Roche Diagnostic Corporation, Indianapolis, IN, USA) in MABS buffer for one hour and then in an AP-coniugated anti-digoxigenin Fab fragment (1:5000 dilution, Roche Diagnostic Corporation, Indianapolis, IN, USA) for one hour. After two washing steps of 30 minutes in MABS buffer and 5 minutes in Buffer 3, the reaction was developed overnight with BCIP/NBT substrate (Sigma-Aldrich, St. Louis, MO, USA). When the color reaction was completed, sections were washed in Buffer 4 and in PBS for some minutes. The slides were examined by means of a Zeiss Axiophot light microscope (Zeiss, Oberkochen, Germany). Micrographs were taken by means of a Nikon Coolpix 950 digital camera (Nikon, Mellville, NY, USA) attached to the same microscope. Images were processed using Adobe Photoshop CS2 9.0.2 (Adobe System, San Josè, CA, USA).

### Real Time RT-PCR analysis of the mouse brain

The quantitative RT-PCR analysis of the transcripts was performed on hemibrains and on dissected brain regions collected from 5 female CD-1 mice (Harlan, Corezzana, Italy) at 6 months of age. The mice were anaesthetized with isoflurane (Isoflurane-vet, Merial, Milan, Italy) and decapitated. Animal procedures were approved by the Animal Care and Use Committee of the University of Torino. The brains were quickly removed from the skull, placed in an ice-cold artificial liquor (containing, in mM: 125 NaCl, 2.5 KCl, 2 CaCl_2_, 1 MgCl_2_, 1.25 NaH_2_PO_4_, 26 NaHCO_3_, 20 glucose, bubbled with 95% O_2_- 5% CO_2_). For the analysis performed on the dissected brain regions, the cerebellum and olfactory bulbs were manually dissected immediately after the whole brain collection. Then, coronal sections from neocortex and hippocampus were cut using a vibratome (Leica Microsystems GmbH, Wetzlar, Germany), starting from Bregma 1.10 mm. First, two 400 μm thick slices were cut in order to collect samples of neocortex. The two hippocampal slices (400 μm thick) were cut starting from Bregma -1.50. White matter parts were manually separated and not included in the gene expression analysis. Stereotaxic coordinates were obtained from the mouse brain atlas "The mouse brain in stereotaxic coordinates" [18]. All samples were rapidly frozen and stored at -80°C.

Total RNA was isolated by extraction with the commercially available TRIzol Reagent (Invitrogen Life Technologies Inc., Grand Island, NY, USA), in accordance with the manufacturer's instructions. Genomic DNA was eliminated by treating the extracted RNA with DNase (Deoxiribonuclease I; Sigma-Aldrich). RNA concentration and purity were evaluated prior and after the DNase treatment by spectrophotometry. The absence of RNA degradation was confirmed by agarose gel electrophoresis. RNA samples were stored at -80°C.

One μg of total RNA was reverse-transcribed to cDNA using the commercial High-Capacity cDNA Archive Kit (Applied Biosystems, Foster City, CA, USA), according to the manufacturer's instructions. Negative controls of the reverse transcription were always performed. cDNA samples were stored at -20°C.

Quantitative Real Time PCR was carried out using the ABI Prism 7000 Sequence Detection System instrumentation (Applied Biosystems). Taqman Gene Expression Assays were purchased from Applied Biosystems to determine the amount of the three target genes (*Afg3l2 *cod. Mm01258204_m1, *Afg3l1 *cod. Mm00475312_m1, *Spg7 *cod. Mm00462651_m1) and the housekeeping gene phosphoglycerate kinase 1 (*Pgk1*, cod. Mm00435617_m1). PCR amplifications were performed on cDNA samples corresponding to a final RNA concentration 200 pg. PCR was performed according to the following reaction conditions: 50°C for 2 minutes, 95°C for 10 minutes, followed by 50 cycles 95°C for 15 seconds alternating with 60°C for 1 minute. Blank controls were performed on each plate.

For the quantitative comparison of the genes, data extracted from each real time RT-PCR run were analysed by means of the 7000 v1.1 SDS instrument software (Applied Biosystems). The CT (Cycle Threshold) was automatically calculated and used to quantify the starting copy number of the target mRNA. The amounts of the target RNA copies were normalized to the endogenous reference *Pgk1*, because it shows a low variation among mouse brain regions [15]. Data were analysed as in Bortone *et al*. [19] to compensate for small differences in the efficiency of amplification among target and housekeeping genes.

For a comparison of the level of expression of *Afg3l2 *and *Spg7 *transcripts in the cerebellum relative to Purkinje cells, the analysis was performed on 8 cerebella and 5 pools (10 cells each) of Purkinje cell (PCs), collected from another set of 11 adult female CD-1 mice (Harlan, Corezzana, Italy) at 6 months of age. The cerebellum was quickly removed under semi-sterile conditions and either rapidly frozen in -80°C 2-methylbutane and stored at -80°C (8 cerebella) or, for slice preparation, placed in the artificial liquor described above, bubbled with 95% O_2_- 5% CO_2_. To collect PCs, parasagittal sections (200 μm thickness) of the vermis of 3 cerebella were prepared using a vibratome. Single PCs were aspirated into micropipettes under visual control; the tips of the micropipettes were broken into empty 0.2 ml Eppendorf tubes. The entire RNA of each cell was retrotranscribed using the High-Capacity cDNA Archive Kit (Applied Biosystems). The cDNA was purified and concentrated using the QIAEX II Gel Extraction Kit (Qiagen, Hilden, Germany). Quantitative PCR and data analysis were performed as described above. For the comparison of the *Afg3l2 *and *Spg7 *transcripts in the cerebellum relative to Purkinje cells, each sample was normalized to the level of the *18S *rRNA (Applied Biosystems, cod.4319413E). Transcript expression differences were statistically evaluated by means of a one way ANOVA test or Student's t-test. A *P *value < 0.05 was considered significant. All results were expressed as mean ± standard error (SE).

## List of Abbreviations

CT: cycle threshold; HSP: hereditary spastic paraplegia; *i*-AAA: intermembrane space mitochondrial **A**TPase **A**ssociated with a variety of cellular **A**ctivities; *m*-AAA protease: matrix mitochondrial **A**TPase **A**ssociated with a variety of cellular **A**ctivities; RT-PCR: reverse-transcriptase polymerase chain reaction; SCA28: spinocerebellar ataxia, type 28

## Authors' contributions

TS and RP carried out the *in-situ *hybridization study. EB and EH carried out and analysed the real time RT-PCR study. CC and AB designed the probes for *in-situ *hybridization and constructed the plasmids containing the gene fragment of interest. AB conceived the study. FT designed and coordinated the study, analysed the *in-situ *hybridization data and wrote the manuscript. CC and AB helped to draft the manuscript. All authors read and approved the final manuscript.

## Supplementary Material

Additional file 1 **Assays for riboprobes specificity**. A: Slot blot assay to exclude cross-hybridisation of the probes; B: Comparison of staining with antisense and sense riboprobes. Click here for file

Additional file 2 **Riboprobe sequences.** Description: sequence of the riboprobes for Spg7, Afg3l1 and Afg3l2, used for in-situ hybridazation. Click here for file
