# Supplementary figures and images for: Mouse brain expression patterns of Spg7, Afg3l1, and Afg3l2 transcripts, encoding for the mitochondrial m-AAA protease
Source: BMC Neurosci. 2010 Apr 28;11:55. doi: 10.1186/1471-2202-11-55 (PMC2880309; doi:10.1186/1471-2202-11-55)

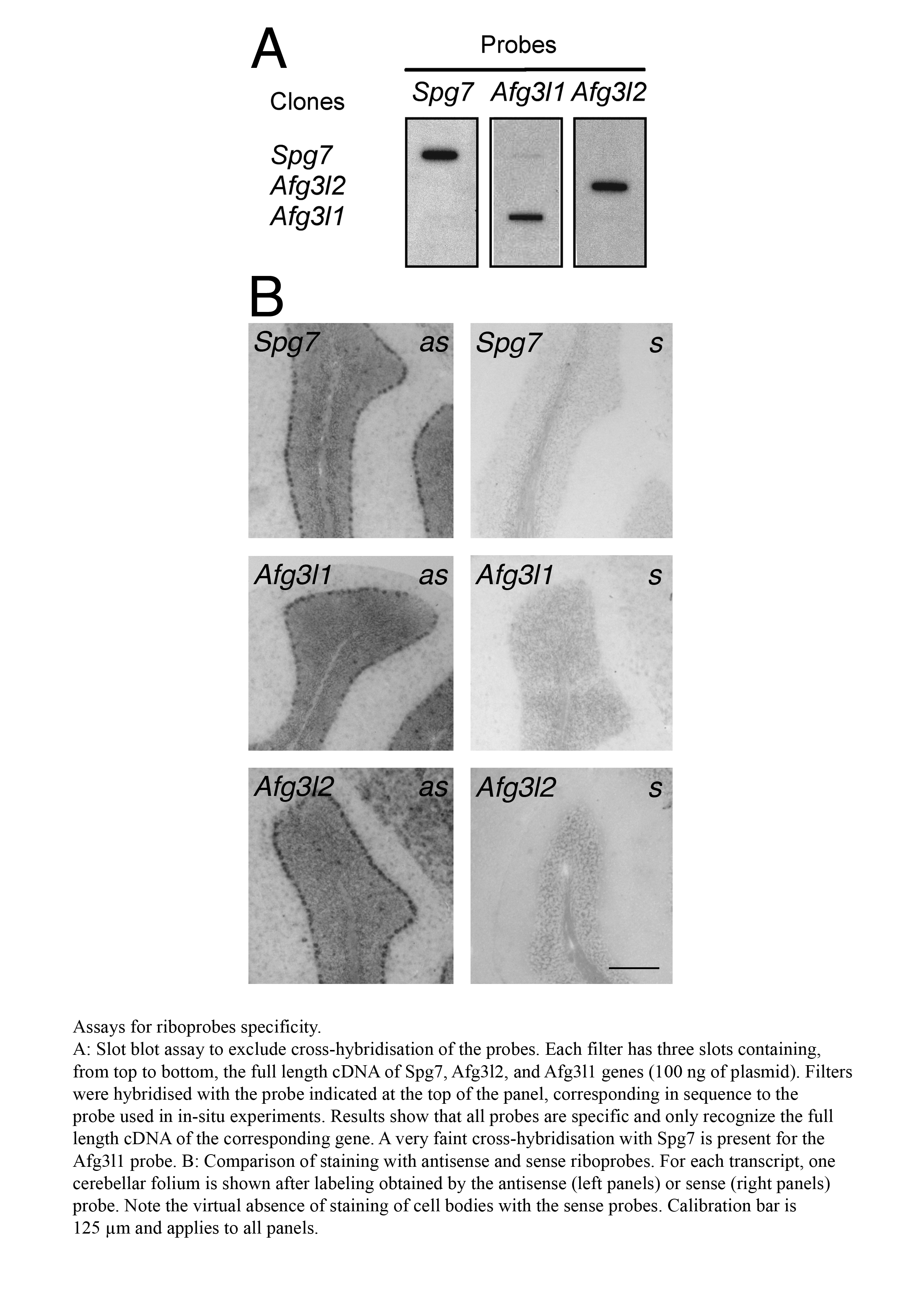

Supplement: Additional file 1 — Assays for riboprobes specificity. A: Slot blot assay to exclude cross-hybridisation of the probes; B: Comparison of staining with antisense and sense riboprobes. [file 1471-2202-11-55-S1.TIFF]
