# Supplementary material for: Mouse brain expression patterns of Spg7, Afg3l1, and Afg3l2 transcripts, encoding for the mitochondrial m-AAA protease
Source: BMC Neurosci. 2010 Apr 28;11:55. doi: 10.1186/1471-2202-11-55 (PMC2880309; doi:10.1186/1471-2202-11-55)
Supplement: Additional file 2 — Riboprobe sequences. Description: sequence of the riboprobes for Spg7, Afg3l1 and Afg3l2, used for in-situ hybridazation. [file 1471-2202-11-55-S2.DOC]

**ADDITIONAL FILE 2**

Riboprobe sequences

*Spg7*

AAGTGGGGGCAGTATTTCCTGGGCTGACTTTGTCAACGAGATGCTGGCTAAAGGCGAGGTGCAGCGTGTGCAGGTGGTGCCCGAGAGTGATGTGGTGGAAGTCTATCTGCATCCTGGAGCTGTGGTGTTTGGGCGGCCTCGGCTGGCCCTGATGTATCGGATGCAGGTTGCAAACATCGACAAATTTGAAGAGAAGCTTCGAGCAGCCGAAGATGAACTGAACATTGAGAGCAAGGACAGGATTCCCGTGTCCTACAAGCGGACAGGATTCTTTGGGAATGCCCTCTACGCCCTGGGGATGACAGCCGTGGGCTTGGCCATCCTGTGGTATGTTTTCAGACTGGCGGGGATGACCGGAAGGGAAGGCGGATTCAGTGCTTTTAATCAGCTTAAGATGGCACGTTTCACCATTGTGGACGGGAAGACAGGGAAAGGAGTCAGCTTCCAAGATGTGGCAGGAATGCATGAAGCCAAGCTGGAAGTCCGAGAATTTGTGGATTATCTGAAGAGCCCAGAGCGTTTCCTTCAGCTCGGTGCCAAGGTTCCAAAGGGTGCCCTGTTGCTGGGGCCCCCTGGCTGTGGGAAGACGCTGTTGGCCAAGGCAGTAGCCACGGAGGCTCAGGTGCCCTTTTTAGCAATGGCTGGCCCAGAGTTTGTGGAGGTGATTGGAGGCCTGGGAGCTGCCCGAGTGCGAAGCCTCTTCAAGGAGGCACGAGCCAGGGCCCCTTGCATAGTGTACATTGATGAGATCGATGCTGTGGGAAAGAAGCGCTCCACCTCCATGTCTGGGTTCTCCAACACGGAAGAGGAGCAGACCCTCAACCAGCTCCTGGTGGAGATGGACGGAATGGGCACCACAGACCATGTCATCGTCTTGGCATCCACCAATCGAGCTGATGTTCTGGACAATGCTCTGATGAGGCCTGGGCGGCTCGACAGGCATGTCTTCATTGATCTTCCCACGCTCCAGGAGAGGCGGGAGATTTTCGAGCAGCACCT

*Afg3l1*

GGAGATGATTTTCCCTGGTGGAAACGGATGCAAAAGGGAGAATTTCCTTGGGACGACAAGGACTTCCGGAGCCTGGCTGTTTTGGGGGCTGGTGTGGCTGCGGGATTTTTATATTTTTATTTCCGAGATCCCGGAAAAGAGATCACCTGGAAACACTTCGTGCAGTATTACCTGGCCAGAGGTCTGGTGGACCGGCTAGAGGTTGTGAACAAGCAGTTTGTGCGTGTTATTCCTGTTCCTGGGACGACATCTGAGAGGTTCGTGTGGTTTAACATTGGCAGTGTTGACACCTTTGAACGGAACCTCGAGTCTGCTCAGTGGGAGCTGGGCATTGAGCCCACCAACCAGGCTGCGGTGGTCTACACTACTGAGAGTGATGGCTCTTTTCTTAGAAGTCTTGTGCCCACTCTGGTCCTGGTTAGCATCCTCCTATATGCTATGAGGAGGGGTCCAATGGGGACTGGTCGCGGTGGGCGAGGAGGAGGCCTCTTCAGTGTTGGTGAGACAACAGCCAAGATCTTAAAGAACAACATCGATGTGCGGTTTGCAGATGTGGCTGGCTGTGAAGAAGCCAAACTGGAAATTATGGAGTTTGTGAATTTCCTGAAGAACCCAAAGCAATATCAGGACTTAGGAGCCAAAATTCCAAAGGGAGCGATGCTCACTGGTCCACCTGGTACTGGCAAAACACTTCTTGCAAAAGCAACTGCTGGGGAGGCCAACGTGCCCTTCATCACCGTGAATGGGTCGGAATT

*Afg3l2*

GAATTCGTGAATTTCTTGAAAAACCCAAAGCAATATCAAGACCTAGGAGCAAAAATCCCAAAGGGTGCCATTCTCACCGGTCCCCCAGGTACTGGGAAGACGCTGCTAGCTAAGGCCACAGCTGGAGAAGCCAATGTCCCCTTTATCACTGTGAGCGGATCTGAGTTTCTGGAGATGTTTGTTGGCGTTGGTCCAGCCAGAGTCCGAGACTTATTTGCCCTTGCTCGGAAGAATGCGCCTTGCATTCTCTTCATTGATGAGATTGATGCTGTGGGAAGGAAGCGCGGCAGAGGCAACTTCGGTGGGCAGAGCGAGCAGGAGAACACACTCAACCAGCTGCTTGTGGAGATGGACGGCTTCAACACAACCACCAATGTGGTCATC.
